# Integrating polygenic risk scores, modifiable dementia risk factors, and sex as predictors of cohort membership in the Alzheimer’s and Lewy body disease spectra: A COMPASS‐ND Study

**DOI:** 10.1002/alz70861_108960

**Published:** 2025-12-23

**Authors:** Sebastian Caballero, Shea J Andrews, Jack H Jhamandas, Richard Camicioli, Mario Masellis, Roger A Dixon

**Affiliations:** ^1^ University of Alberta, Edmonton, AB Canada; ^2^ University of California, San Francisco, San Francisco, CA USA; ^3^ University of Toronto, Toronto, ON Canada

## Abstract

**Background:**

Polygenic risk scores (PRSs) summarize genetic risk across single nucleotide polymorphisms (SNPs). Alzheimer’s disease (AD) and Lewy body disease (LBD) PRSs contribute to risk detection via both multi‐mechanism (SNPs from multiple mechanistic pathways) and mechanism‐specific (SNPs from separate mechanistic pathways) versions. Machine learning techniques compared the relative importance of 14 PRSs and 14 AD/LBD risk factors in discriminating cohorts of AD and LBD spectra, disaggregated by sex.

**Methods:**

Data from the Comprehensive Assessment of Neurodegeneration and Dementia database of the Canadian Consortium on Neurodegeneration in Aging included 7 cohorts (*n*=899; *M* age=72; 49%F). The common benchmark was the cognitively unimpaired (CU) cohort. AD spectrum cohorts: subjective cognitive impairment (SCI), mild cognitive impairment (MCI), and AD. LBD spectrum cohorts: Parkinson’s disease (PD), PD‐MCI, and LBD. PRSice‐2 calculated two multi‐mechanism PRSs (AD‐related, 387 SNPs; LBD‐related, 379 SNPs) and 12 mechanism‐specific PRSs (5‐17 SNPs/mechanism). AD‐specific PRSs: amyloid‐beta metabolism, tau metabolism, lipid metabolism, mitochondrial function, immune response, nervous function, and basic cellular processes. LBD‐specific PRSs: alpha‐synuclein metabolism, lysosomal degradation, ceramide metabolism, dopamine metabolism, and oxidative stress response. We assessed *APOE*+/‐ PRSs. All comparisons included 14 risk factors: demographic (e.g., age), mobility (e.g., gait), lifestyle (e.g., physical activity), biomarkers (e.g., homocysteine), vascular (e.g., pulse pressure), and functional (grip strength). Random forest classifier models tested predictions across spectra, disaggregated by sex.

**Selected Results:**

Overall, we observed in each spectrum (a) strong discrimination across cohorts, (b) varying leading predictors of same‐spectra cohorts, and (c) different prediction patterns by sex. Example for AD spectrum: four leading predictors of SCI/AD in females were multi‐mechanism AD‐related (*APOE*) PRS, gait, age, and education (AUC=.81), whereas four leading predictors in males were amyloid‐beta metabolism (*APOE*) PRS, lipid metabolism (*APOE*) PRS, LDL cholesterol, and vitamin B12 (AUC=.92). Example for LBD spectrum: four leading predictors of PD‐MCI/LBD in females were multi‐mechanism LBD‐related PRS, ceramide metabolism PRS, education, and homocysteine (AUC=.77), whereas five leading predictors in males were multi‐mechanism LBD‐related (*APOE*) PRS, lysosomal degradation PRS, age, nutrition, and total bilirubin (AUC=.84).

**Conclusion:**

Integration of neurodegenerative disease‐related PRSs and risk factors promotes precision identification of sex‐specific cohort discrimination in the AD and LBD spectra.

